# A Telementoring Program and Hepatitis C Virus Care in Rural Patients

**DOI:** 10.1089/tmr.2021.0001

**Published:** 2021-05-13

**Authors:** Ping Du, Xin Yin, Lan Kong, Jeah Jung

**Affiliations:** ^1^ Department of Medicine, College of Medicine, The Pennsylvania State University, Hershey, Pennsylvania, USA.; ^2^ Department of Public Health Sciences, College of Medicine, The Pennsylvania State University, Hershey, Pennsylvania, USA.; ^3^ Department of Health Policy and Administration, College of Health and Human Development, The Pennsylvania State University, University Park, Pennsylvania, USA.

**Keywords:** HCV care, hepatitis C virus (HCV), telementoring

## Abstract

**Background:** Rural patients with chronic hepatitis C virus (HCV) infection may be less likely to access HCV care than those in urban areas. A telementoring, task-shifting model has been implemented to address the unmet needs of HCV care. Evidence is needed on whether this intervention improves HCV care in rural HCV patients.

**Methods:** We compared three key HCV care indicators among Medicare patients with chronic hepatitis C in 2014–2016 by urban–rural status between New Mexico with a telementoring program and Pennsylvania without such a program. We classified each patient's urban–rural status based on his or her ZIP code of residence. We used multivariable log-binomial regressions to examine the relative probability of receiving HCV care by urban and rural status in two states.

**Results:** In New Mexico, 41.3% of HCV patients resided in rural areas (*N* = 1155). In Pennsylvania, rural patients accounted for 13.2% (*N* = 1775). The unadjusted overall rates of receiving HCV RNA or genotype testing within 12 months before HCV treatment were 76.1% in “rural-New Mexico” versus 73.3% in “rural-Pennsylvania,” 66.2% in “urban-New Mexico,” and 70.2% in “urban-Pennsylvania.” Post-treatment HCV RNA testing rate was also high in “rural-New Mexico” (83.0%). After adjusting for demographic and clinical characteristics, “rural-New Mexico” HCV patients who received HCV treatment still had the highest probability of taking HCV RNA or genotype testing before HCV treatment, compared with other groups (relative risk [95% confidence interval]: 0.91 [0.84–1.00] in “rural-Pennsylvania,” 0.85 [0.78–0.93] in “urban-New Mexico,” and 0.93 [0.87–1.00] in “urban-Pennsylvania”).

**Conclusions:** The telementoring program may help improve HCV care in rural patients.

## Introduction

Patients with chronic hepatitis C virus (HCV) infection have increased risks of developing liver and extrahepatic complications.^1^ To encourage routine HCV care, the Centers for Medicare and Medicaid Services (CMS) has included specific HCV care quality measures in its Physician Quality Reporting System (PQRS).^2^ However, rural HCV patients may be less likely to access HCV care due to the lack of specialists in rural areas or logistic barriers, compared with urban HCV patients.^3–7^ Various interventions have been developed to address the unmet needs of HCV care in rural populations, but the effect of these interventions remains unclear.^8–12^

Developed in 2003, the Project Extension for Community Healthcare Outcomes aims to use telementoring, a guided practice model where primary care phycisians (PCPs) keep managing patients through tele-education with specialists at academic health centers, to help PCPs in rural areas of New Mexico manage HCV patients.^13–15^ In our previous research, we found that a telementoring, task-shifting model helped reduce urban–rural disparities in HCV treatment possibly through educating rural primary care providers to accumulate knowledge and confidence in using highly effective and well-tolerated direct-acting antiviral agents (DAAs) to treat HCV patients.^16^ In this study, we further evaluated if that telementoring model, which was designed to address provider shortages in rural areas, also improved key HCV care indicators in rural HCV patients.

## Methods

We used the U.S. Medicare Fee-For-Service claims and outpatient prescription drug (Part D) data in 2014–2017 to examine HCV care indicators among Medicare patients with chronic HCV infection. We focused on patients who newly sought HCV care in 2014–2016, defined as having at least one HCV-related inpatient claim or two outpatient claims in 2014–2016, but no HCV-related claims during the previous 12 months. We identified the patient cohorts in each year using the International Classification of Disease diagnosis codes for chronic HCV in the claims and followed all patients till loss to follow-up (switch to Medicare Advantage or disenrollment of Part D), death, or the end of 2017.

The study outcomes were three key HCV care indicators that were included in the CMS PQRS and/or that were recommended by the medical communities (such as American Association for the Study of Liver Diseases): quantitative HCV RNA or genotype testing conducted within 12 months before the initiation of HCV DAA treatment (representing linkage to HCV treatment); quantitative HCV RNA testing after DAA treatment (indicating HCV treatment monitoring); and screening for hepatocellular carcinoma (HCC) with ultrasound, contrast-enhanced computed tomography, or magnetic resonance imaging for cirrhotic patients (reflecting liver disease management).^17–19^

We compared these HCV care indicators between urban and rural HCV patients in two states: New Mexico with a widely implemented telementoring program to train rural PCPs to deliver HCV care, and Pennsylvania without a similar intervention targeting rural populations. We assigned each patient's urban–rural status based on his/her ZIP code of residence using the 2006 ZIP code level Rural–Urban Commuting Area codes (1–3: urban, 4–10: rural).^20^ We reported the numbers and percentages of HCV patients receiving each HCV care in each cohort year by urban–rural status and by state (urban-New Mexico, urban-Pennsylvania, rural-New Mexico, and rural-Pennsylvania). We also performed multivariable log-binomial regressions with all cohorts combined to estimate relative risks (RRs) and their 95% confidence intervals (CIs) of HCV care indicators while adjusting for patients' demographic and clinical characteristics (age, sex, race/ethnicity, receiving a low-income subsidy, liver cirrhosis status, drug use disorder, Charlson comorbidity index, and cohort year). We used SAS 9.4 (SAS Institute, Cary, NC) to analyze all the data and evaluated statistical significance based on the Wald chi-square test at a two-sided *p*-value <0.05. The Pennsylvania State University Institutional Review Board approved this study (IRB#: STUDY00007907) and informed consent was not required for this study.

## Results

Table 1 shows that in New Mexico, 41.3% of HCV patients resided in rural areas (*N* = 1155), but in Pennsylvania, rural patients accounted only for 13.2% (*N* = 1775). The patients' characteristics regarding race, socioeconomic status, and clinical conditions were different between New Mexico and Pennsylvania: HCV patients in “rural-New Mexico” were more likely to be nonwhites and older, receive low-income subsidy, and have decompensated cirrhosis, compared with HCV patients in “rural-Pennsylvania.”

**Table 1.** Selected Characteristics of Hepatitis C Virus Patients by Urban and Rural Status in Two States

Characteristics	Rural		Urban		Total (*N* = 16,266)
	New Mexico (*n* = 1155)	Pennsylvania (*n* = 1775)	New Mexico (*n* = 1639)	Pennsylvania (*n* = 11,697)	
Age (mean [SD])	58.9 (11.5)	57.6 (12.5)^**^	59.0 (11.9)	60.3 (11.2)^***^	59.8 (11.5)
Female (%)	32.4	35.3	38.1^**^	35.0	35.2
White (%)	74.0	91.4^***^	74.1	61.0^***^	66.6
Low-income subsidy (%)	67.9	63.0^**^	67.4	65.3	65.5
Drug/alcohol use (%)	50.4	55.0^*^	51.9	50.2	50.9
Decompensated cirrhosis (%)	11.5	8.3^**^	10.3	7.8^***^	8.4
Charlson comorbidity index ≥3 (%)	48.3	49.3	44.1^*^	54.1^**^	52.2

Chi-square tests or Student's *t*-tests, *p*-values: ^*^p < 0.05, ^**^p < 0.01, ^***^p < 0.0001 (Referent group: rural-New Mexico patients).

SD, standard deviation.

Table 2 presents descriptive data on three key HCV care indicators by urban–rural status in each state. For HCV RNA or genotype testing 12 months before DAA treatment among DAA users, “rural-New Mexico” showed the highest unadjusted rate across nearly all cohort years (the overall unadjusted rate: 76.1% in “rural-New Mexico” versus 73.3% in “rural-Pennsylvania,” 66.2% in “urban-New Mexico,” and 70.2% in “urban-Pennsylvania”). HCV RNA testing rates after DAA treatment were also high in “rural-New Mexico” patients (the overall unadjusted rate: 83.0% in “rural-New Mexico” versus 82.8% in “rural-Pennsylvania,” 75.5% in “urban-New Mexico,” and 77.9% in “urban-Pennsylvania”). In addition, the urban–rural differences in use of these two HCV care indicators were greater in New Mexico than in Pennsylvania. However, regarding HCC screening among cirrhotic patients, the urban groups showed higher rates of use than rural groups, and “urban-New Mexico” showed the highest proportion of cirrotic patients who received HCC screening (53.1%).

**Table 2.** Hepatitis C Virus Care Indicators by Urban and Rural Status in Two States

HCV care indicator by cohort year	Rural		Urban		Total, %
	New Mexico, *n* (%)	Pennsylvania, *n* (%)	New Mexico, *n* (%)	Pennsylvania, *n* (%)	
Total	1155	1775	1639	11,697	16,266
HCV RNA viral load or genotype test within 12 months before DAA treatment among DAA users					
2014	84 (77.4)	166 (72.3)	181 (65.7)	1290 (74.7)	73.7
2015	95 (80.0)	142 (78.9)	175 (64.6)	1073 (66.8)	68.5
2016	68 (69.1)	104 (67.3)	114 (69.3)	731 (67.2)	67.6
All cohorts	247 (76.1)	412 (73.3)	470 (66.2)	3094 (70.2)	70.4
Post-treatment HCV RNA viral load test among DAA users					
2014	84 (82.1)	166 (83.7)	181 (70.2)	1290 (76.9)	77.1
2015	95 (84.2)	142 (83.8)	175 (80.6)	1073 (79.8)	80.6
2016	68 (82.4)	104 (79.8)	114 (76.3)	731 (76.7)	77.4
All cohorts	247 (83.0)	412 (82.8)	470 (75.5)	3094 (77.9)	78.4
Any HCC screening among cirrhotic patients					
2014	147 (34.0)	168 (41.7)	163 (52.8)	1155 (45.3)	44.7
2015	99 (46.5)	126 (41.3)	118 (49.2)	703 (46.1)	45.9
2016	76 (36.8)	76 (50.0)	92 (58.7)	429 (48.3)	48.6
All cohorts	322 (38.5)	370 (43.2)	373 (53.1)	2287 (46.1)	45.8

DAA, direct-acting antiviral agent; HCC, hepatocellular carcinoma; HCV, hepatitis C virus.

Table 3 reports results from multivariable log-binomial regressions. After adjusting for age, sex, race/ethnicity, receiving a low-income subsidy, liver cirrhosis status, drug use disorder, Charlson comorbidity index, and cohort year, “rural-New Mexico” DAA users still had the highest likelihood of taking HCV RNA or genotype testing 12 months before DAA treatment, when compared with other groups (RR [95% CI]: 0.91 [0.84–1.00] in “rural-Pennsylvania,” 0.85 [0.78–0.93] in “urban-New Mexico,” and 0.93 [0.87–1.00] in “urban-Pennsylvania”). While “rural-New Mexico” DAA users performed better than “urban-New Mexico” for post-treatment HCV RNA viral load test (“urban-New Mexico,” RR [95% CI]: 0.92 [0.85–0.99]), “urban-New Mexico” cirrotic patients had higher likelihood of receiving HCC screening (“urban-New Mexico,” RR [95% CI]: 1.37 [1.16–1.62]).

**Table 3.** Comparison of Hepatitis C Virus Care Indicators by Urban and Rural Status in Two States Using Multivariable Log-Binomial Regressions

	Rural			Urban			
		Pennsylvania (*n* = 1775)		New Mexico (*n* = 1639)		Pennsylvania (*n* = 11,697)	
	New Mexico (*n* = 1155)	RR (95% CI)	*p*	RR (95% CI)	*p*	RR (95% CI)	*p*
HCV RNA viral load or genotype test within 12 months before DAA treatment among DAA users							
All cohorts	Reference	0.91 (0.84–1.00)	0.0396	0.85 (0.78–0.93)	0.0004	0.93 (0.87–1.00)	0.0426
Post-treatment HCV RNA viral load test among DAA users							
All cohorts	Reference	1.01 (0.94–1.08)	0.8831	0.92 (0.85–0.99)	0.0205	0.98 (0.92–1.03)	0.4220
Any HCC screening among cirrhotic patients							
All cohorts	Reference	1.08 (0.90–1.30)	0.4057	1.37 (1.16–1.62)	0.0002	1.15 (0.99–1.34)	0.0631

Results are from log-binomial models adjusting for age, sex, race/ethnicity, LIS, liver cirrhosis, drug use disorder, Charlson comorbidity index, and cohort year. *p*-Values are based on Wald chi-square tests.

CI, confidence interval; LIS, receiving a low-income subsidy; RR, relative risk.

## Discussion

We found that two HCV care indicators (pre- or post-treatment quantitative HCV RNA or genotype tests) among rural HCV patients in New Mexico, where a telementoring program was available, outperformed urban patients in New Mexico and patients living in Pennsylvania without such a program. In response to the HCV epidemic and a shortage of specialists in rural areas, a telementoring-based, task-shifting intervention has been implemented since 2003 in New Mexico to train rural PCPs by experienced hepatologists to treat HCV infection.^13–15^ In recent years, with the availability of highly effective HCV DAAs (especially new pan-genotypic DAAs with broader indications), a simplified HCV management model has been proposed for PCPs to provide HCV care.^19^ Because one of the main goals of HCV care programs was to improve HCV treatment, the better performance of two HCV treatment-related indicators among rural HCV patients in New Mexico may reflect that the telementoring intervention has resulted in an intended outcome.

However, about 30% of patients did not receive HCV RNA or genotype testing 12 months before DAA treatment in both states, which is unexpected as these tests might be necessary to get prior authorization before treatment. Moreover, HCC screening among cirrhosis patients, an indicator of liver disease management that requires a referral to a specialist, remained low among rural patients in both states.^19^ This finding reflects the constant challenge of the lack of speciality care in rural areas. As cirrhotic patients are at a higher risk of developing liver cancer or other complications, continuous monitoring of their liver disease status should be included as a part of future interventions to help PCPs improve HCV care for rural patients.

Several important limitations need to be recognized in interpreting the study results. While the telementoring intervention has been well established in New Mexico, we could not identify HCV patients cared by PCPs who participated in the intervention. Some urban primary care providers may also have participated in the telementoring intervention. Meanwhile, Pennsylvania had several large medical centers serving rural or underserved populations, and so, local interventions to improve HCV care in rural populations may have been implemented. Our analysis did not fully account for those factors. Second, we could not assess other recommended HCV care indicators (such as hepatitis A vaccination, review of current medication list, review of treatment option, and liver fibrosis stage) because of the general limitations of claims data. Third, our results do not imply a causal effect and the observed differences may be partially due to differences in other health service use between the two states. Finally, our results only reflected the possible intervention effects in Medicare patients and the clinical implications of our study findings remain to be explored.

In conclusion, our study suggests that the telementoring, task-shifting intervention may be an effective strategy to help improve HCV care among rural patients. Future interventions need to address liver disease management by PCPs to reduce HCV-related adverse health outcomes for rural patients.

## Abbreviations Used

CI: confidence interval
CMS: Centers for Medicare and Medicaid Services
DAA: direct-acting antiviral agent
HCC: hepatocellular carcinoma
HCV: hepatitis C virus
LIS: receiving a low-income subsidy
PCPs: primary care phycisians
PQRS: Physician Quality Reporting System
RR: relative risk
SD: standard deviation
